# Record of Medical Science

**Published:** 1846-04

**Authors:** 


The disease which has proved so destructive among the cattle in the southern provinces of Russia, is said to present all the symptoms of the cholera. Those who remember the progress of that scourge in 1830–31, state that the same phenomena are met with in animals labouring under the present epidemic, as were then seen in persons affected with the cholera.—*Ibid.*

